# Cuticular Waxes of *Arabidopsis thaliana* Shoots: Cell-Type-Specific Composition and Biosynthesis

**DOI:** 10.3390/plants6030027

**Published:** 2017-07-07

**Authors:** Daniela Hegebarth, Reinhard Jetter

**Affiliations:** 1 Department of Botany, University of British Columbia, 6270 University Boulevard, Vancouver, BC V6T 1Z4, Canada; daniela.hegebarth@botany.ubc.ca; 2 Department of Chemistry, University of British Columbia, 2036 Main Mall, Vancouver, BC V6T 1Z1, Canada

**Keywords:** trichomes, cuticular wax, chain length, *Arabidopsis thaliana*, KCS, elongation, fatty acid elongase complex, ketoacyl-CoA synthase

## Abstract

It is generally assumed that all plant epidermis cells are covered with cuticles, and the distinct surface geometries of pavement cells, guard cells, and trichomes imply functional differences and possibly different wax compositions. However, experiments probing cell-type-specific wax compositions and biosynthesis have been lacking until recently. This review summarizes new evidence showing that *Arabidopsis* trichomes have fewer wax compound classes than pavement cells, and higher amounts of especially long-chain hydrocarbons. The biosynthesis machinery generating this characteristic surface coating is discussed. Interestingly, wax compounds with similar, long hydrocarbon chains had been identified previously in some unrelated species, not all of them bearing trichomes.

## 1. Introduction

All above-ground plant parts, in the primary state of development, are lined by a layer of epidermis cells that serve crucial functions for protecting the various organs and, thus, for plant survival. The epidermis consists of three different cell types, the pavement cells, guard cells, and trichomes, in characteristic numbers, shapes and geometric arrangements depending on the species, organ, and developmental state. 

**Figure 1:**
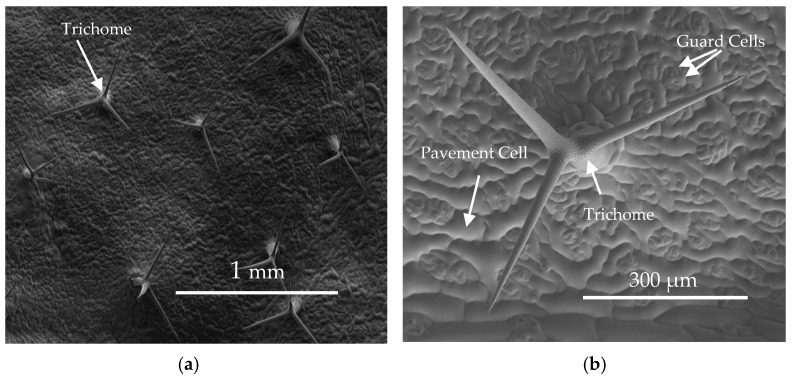
Cryo-SEM images of abaxial *Arabidopsis* leaf surfaces. (**a**) Comparison of abundance and size of pavement and guard cells covering the leaf surface, relative to the trichome cells protruding out of the surface; (**b**) Detailed view of a single trichome, showing its shape and cell size relative to pavement and guard cells (reprinted from [6,7] with permission).

The different epidermis cell types serve very different functions (Figure 1; Table 1): pavement cells form the major protective surface barrier [1], and mature *Arabidopsis* leaves contain about 29,000 cells each with a surface area of ca. 4000 μm^2^ [2] (Table 1). Guard cells, on the other hand, are important for regulating gas exchange and for protecting the surface around stomata [3]. They are less abundant and smaller than pavement cells (Figure 1b), with ca. 10,000 guard cells on average per *Arabidopsis* leaf and average sizes of about 280 μm^2^ [2,4] (Figure 1b, Table 1). Finally, trichomes emerge vertically out of the surface (Figure 1a), serving a variety of roles including UV protection, heat insulation, transpiration control, and insect deterrence [5]. *Arabidopsis* trichomes consist of a stalk with two or three perpendicular arms (Figure 1a,b), and they are far less abundant (about 75 trichomes per leaf) but larger (surface areas about 40,000 μm^2^) than the other epidermal cell types [2] (Table 1).

**Figure 2:**
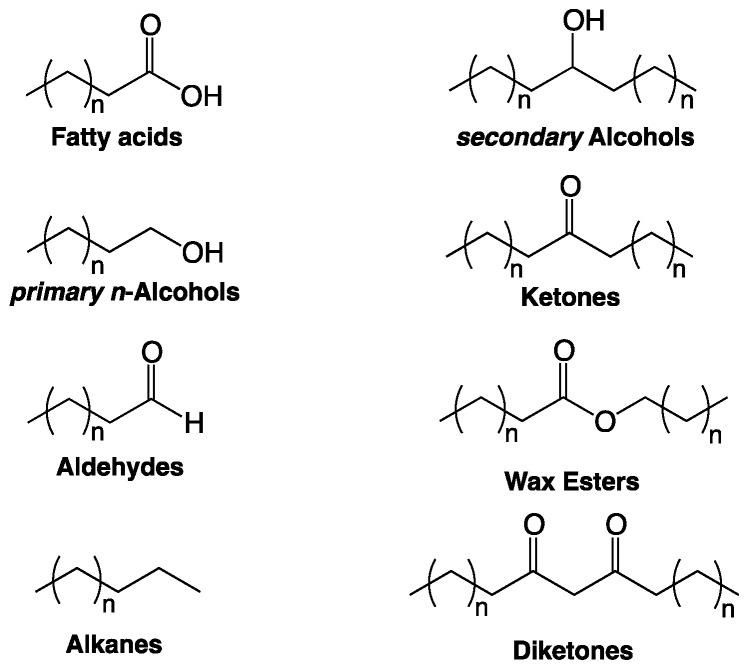
Chemical structures of major cuticular wax compound classes.

It is generally assumed that all three epidermal cell types are covered with an uninterrupted cuticle, a hydrophobic surface consisting of a cutin matrix [8] and solvent-soluble waxes embedded in, and deposited onto, it. Cutin is a polyester of saturated and unsaturated C_16_ and C_18_ ω-hydroxyacids, polyhydroxyacids, or epoxyacids and glycerol [9,10]. Cuticular wax usually comprises a variety of aliphatic compound classes such as fatty acids, primary *n*-alcohols, secondary alcohols, alkyl esters, aldehydes, and alkanes, but also polyketides and terpenoids (Figure 2). Within the compound classes, usually compounds with varying carbon numbers in the hydrocarbon chains are found, thus defining series of aliphatic homologs. Both the abundances of individual constituents within the wax mixture and the relative amounts of wax and cutin vary greatly between plant species, organs, and developmental stages. For instance, in leaf blades of *Triticum aestivum* seedlings, primary *n*-alcohols are the predominant compound class, whereas on flag leaf sheaths β-diketones are predominant [11]. *Arabidopsis* leaf wax contains alkanes with a broad chain length ranging from C_25_ to C_34_, while *Arabidopsis* stem wax consists mainly of C_29_ alkane.

**Figure 3:**
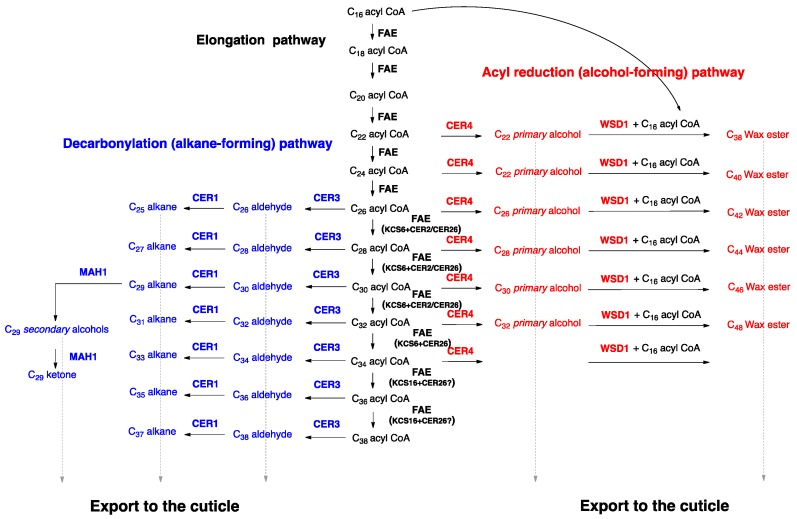
Wax biosynthesis pathways in *Arabidopsis*. First, acyl-CoAs are elongated by fatty acid elongase (FAE) complexes (black), then their head groups are modified along either the alkane-forming pathway (blue) or the alcohol-forming pathway (red), and the wax compounds are exported to the cuticle (dashed gray arrows).

The mechanisms underlying wax biosynthesis have been largely elucidated using model organisms such as *Arabidopsis* and tomato. First, C_16_ and C_18_ fatty acid thioesters are synthesized de novo in the plastids of epidermal cells. These precursors are then hydrolyzed to free acids, exported to the endoplasmic reticulum (ER), and activated to acyl-CoAs by long chain acyl-CoA synthases (LACSs) [12]. At the ER, acyl-CoAs are elongated in several elongation cycles from C_16_ and C_18_ to very-long-chain fatty acids (VLCFA), which usually have aliphatic chains with 24–34 carbons [13] (Figure 3). 

**Figure 4:**
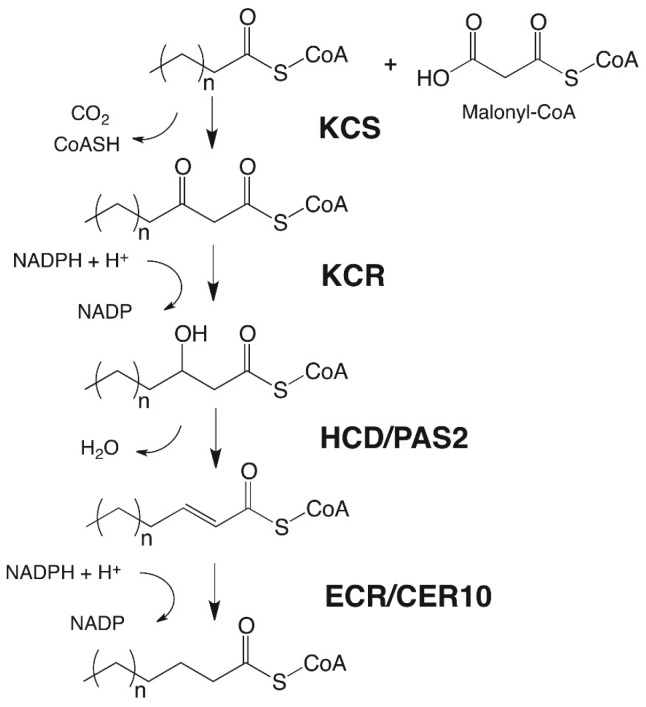
Elongation of acyl-CoAs by the fatty acid elongase (FAE) complex.

Each elongation cycle is carried out by a fatty acid elongase (FAE), an enzyme complex catalyzing four sequential reactions effecting the overall extension of the hydrocarbon chain by two carbons (Figure 3 and Figure 4). In each cycle, first a β-ketoacyl-CoA synthase (KCS) fuses the incoming chain with a C_2_ unit from malonate, and then a β-ketoacyl-CoA reductase (KCR), a β-hydroxyacyl-CoA dehydratase (HCD), and an enoyl-CoA reductase (ECR) reduce the functional group of the intermediate into a methylene (CH_2_) unit [14,15,16,17,18,19,20] (Figure 4). The initial condensing reaction, catalyzed by KCS enzymes, is the rate-limiting step and determines the chain length range of substrates and products of the FAE complex [19], while the other three FAE enzymes, the KCR, HCD, and ECR, are used ubiquitously by all FAE complexes [19,21]. It is likely that different FAEs co-exist within one cell, together generating a broad chain length range of acyls with predominantly even carbon numbers. Based on *Arabidopsis ksc* mutant analyses, the KCS enzyme KCS6/CER6 was found to be central for cuticular wax biosynthesis, as it elongates C_24_ to C_28_ acyl-CoAs. More recently, it was shown that the KCS6/CER6 FAE complex may be associated with CER2-LIKE proteins, then enabling elongation of acyl-CoAs up to C_34_ (Figure 3), yet the mechanism of action of CER2-LIKE proteins remains unknown [22,23,24,25].

After elongation, acyl-CoAs are modified on two branch pathways: on the alkane-forming pathway, the CER3 and CER1 enzymes consecutively reduce and decarbonylate acyls, into aldehydes with predominantly even carbon numbers, and alkanes one carbon shorter, and thus odd C-numbers, respectively [26,27,28] (Figure 3). The alkanes may be converted into secondary alcohols and ketones by the mid-chain alkane hydroxylase1 (MAH1) [29]. On the alcohol-forming pathway, fatty acyl-CoA reductases (FARs) form even-numbered *n*-alcohols [30], which can be converted into alkyl esters by the wax synthase/diacylglycerol acyltransferase1 (WSD1) enzyme [31] (Figure 3).

It is well established that cuticular waxes are the crucial component of the cuticle, serving its primary physiological function as a barrier that limits transpirational water loss [32]. Therefore, it is generally assumed that all three epidermal cell types must be lined by continuous cuticles comprising similar wax mixtures to protect the entire plant tissue. However, it is not clear whether the pavement cells, guard cells and trichomes have autonomous wax biosynthesis machineries, and whether they produce different wax mixtures to serve slightly different functions in the different geometric contexts of the three cell types. Indirect evidence had gradually accumulated, suggesting that at least trichomes may have cuticular wax compositions and biosynthesis distinct from those of pavement cells, and very recently also direct evidence on trichome waxes has emerged. The current review will summarize all this evidence, focusing first on the composition of *Arabidopsis* trichome waxes (Section 2) and the trichome-specific wax biosynthesis mechanisms in *Arabidopsis* (Section 3), and then providing context on similar wax compositions in other plant species (Section 4), as well as their possible implications on wax structure, properties, and functions (Section 5).

## 2. Composition of Cuticular Wax Covering *Arabidopsis* Trichomes 

Several lines of evidence have addressed the question whether cuticular wax on *Arabidopsis* trichomes differs from that on pavement cells, first using indirect approaches and, in recent years, also direct chemical analyses.

Initially, indirect evidence came from SEM investigations of *Arabidopsis* stem surfaces. It had repeatedly been reported that the surface of stem pavement cells is covered with epicuticular wax crystals [33,34,35,36,37], while the surface of stem trichomes was devoid of crystals [6,7,38]. It is well established that epicuticular wax crystals form due to the accumulation of one or a few compounds in the wax mixture, and the absence or presence of crystals, hence, reflects different amounts of the crystal-forming compounds within the overall wax mixture [39,40]. Consequently, the different micro-reliefs of *Arabidopsis* stem pavement and trichome surfaces suggested that these two cell types have different wax compositions. However, this qualitative comparison did not reveal how exactly both wax mixtures differ and, therefore, additional information was required. Unfortunately, such evidence could not be drawn from analogous SEM studies of other *Arabidopsis* organ surfaces, because they either lack trichomes or else their pavement and trichome cells do not differ in surface morphology. Other, more quantitative approaches were necessary to gauge the differences between the trichome and pavement wax mixtures.

**Figure 5:**
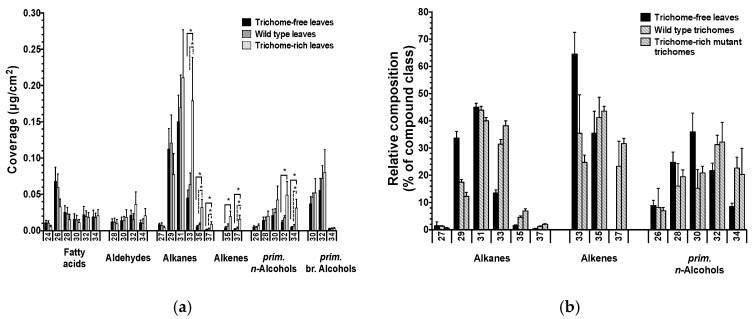
Wax composition of *Arabidopsis* leaves and isolated trichomes. (**a**) Coverage of single wax compounds within each compound class on leaves of the trichome-free mutant *gl1*, the wild type, and the trichome-rich mutant *cpc tcl1 etc1 etc3*; (**b**) Relative distributions of single compounds within each compound class on leaves of the trichome-free mutant *gl1*, on trichomes isolated from wild type leaves, and on trichomes isolated from trichome-rich mutant leaves. Average values are given with standard deviations (*n* = 5). The *x*-axis labels indicate the total carbon chain numbers of compounds. Asterisks indicate discovery of significant differences between coverages based on Student’s *t*-test (* = *p* < 0.05) (adapted from [6]).

First quantitative distinctions between pavement and trichome surface compositions were enabled by *Arabidopsis* mutant studies [6]. In these experiments, compositions of cuticular waxes were compared between the trichome-free *gl1* mutant, the wild type, and the trichome-rich mutant *cpc tcl1 etc1 etc3*. The leaf wax of the trichome-rich mutant contained higher amounts of C_32_–C_37_ wax compounds compared to the wild type and trichome-free mutant (Figure 5a). The shifts in chain length distributions occurred within all major compound classes, irrespective of their functional groups, whereas the absolute amounts of the compound classes were not significantly different between lines with different trichome numbers. Moreover, the stem waxes of the trichome mutant lines showed similar effects to those observed for leaves, albeit with increases mainly in C_32_ and C_33_ compounds [6]. Overall, these results revealed a correlation between the abundances of trichomes and extremely long-chain aliphatic compounds. Pavement and trichome waxes were, thus, found to differ in their chain length profiles.

The inferences from mutant comparisons were confirmed by further investigations into wax compositional changes on developing Arabidopsis wild type leaves [2]. It had previously been shown that, in most dicot species, trichome development starts very early during leaf ontogeny, before major pavement cell expansion. Therefore, immature (still expanding) leaves have higher trichome:pavement surface ratios than mature leaves, and trichomes constitute a larger portion of the surface area in younger leaves. Comparisons of cuticular wax compositions between young and mature leaves can thus be interpreted as proxies for differences in cuticular wax composition of trichomes and pavement cells. Most interestingly, young wild type leaves had relatively high abundances of C_35+_ compounds, which then decreased over the course of leaf development. This shift in chain length profiles was accompanied by a steady decrease of apparent wax coverages, calculated as wax amounts relative to the macroscopic leaf surface and, thus, likely reflecting the steady decrease in microscopic aspect ratios due to declining trichome:pavement ratios. Taken together, the time-dependent wax compositional changes suggested that the levels of C_35+_ compounds were positively correlated with trichome densities, confirming that trichome and pavement waxes had different chain length profiles.

To further corroborate the conclusions from the previous mutant and time course studies, both approaches were combined to monitor the wax development of the trichome-less *Arabidopsis* mutant *gl1* [2]. In contrast to wild type, *gl1* leaf wax coverages did not change during development, and in this mutant C_35+_ compounds were detected at relatively low, constant levels throughout leaf expansion. These results were in stark contrast to the previously observed apparent drop in both wax coverage and C_35+_ compounds during wild type leaf expansion, thus confirming that both effects depended on the presence of trichomes. The mutant time-course data further underline the conclusion that trichome surfaces are distinguished from those of pavement cells through the higher abundance of compounds with extremely long aliphatic chains.

Finally, the cell-type-specific surface compositions were assessed directly by chemical analyses of waxes from isolated trichomes [6]. The selectively sampled wax from *Arabidopsis* leaf trichomes had higher amounts of C_32+_ compounds than pavement cell wax (as judged by the composition of the *gl1* leaf wax) (Figure 5b), thus confirming the results from the previous mutant comparisons and time course studies. Interestingly, the direct analysis of wax from isolated (leaf) trichomes also revealed that it contained mainly alkanes and primary *n*-alcohols, only two of the many compound classes found in pavement wax. The trichome wax further comprised small amounts of alkenes, a compound class not reported for *Arabidopsis* waxes before. It seems likely that the alkenes had not been noticed in earlier studies, due to detection problems caused by relatively small trichome contributions to total wax extracts from mature leaves. 

In summary, all the recent indirect and direct evidence led to matching conclusions, showing that the cuticular wax lining Arabidopsis trichomes differs significantly from that on pavement cells. Trichome wax is distinguished by a relatively simple composition with only few compound classes, by the presence of alkenes, and by shifts to longer chain lengths in all compound classes. 

With such differences between Arabidopsis cell types now firmly established, we must consider whether the compositional gradients between trichomes and adjacent pavement might lead to lateral diffusion along the surface of these cells. To address possible lateral exchange of constituents, the mobility of compounds within wax mixtures must be assessed. Schreiber [41] determined self-diffusion coefficients of VLC compounds within wax, reaching values of approximately 10^−20^ m^2^ s^−1^ for example for C_24_–C_28_ compounds. Based on these self-diffusion coefficients, we predict that wax molecules will not migrate fast enough within the mixture to cause significant exchange of material between trichome and pavement waxes. Moreover, if diffusion were to occur, it would only lessen differences over time rather than enhancing them. Consequently, the observed differences in wax compositions of both cell types at certain times after cuticle formation must be regarded as minimum effects and, assuming limited, local migration, even more drastic differences may have been initially established during trichome development.

## 3. Wax Biosynthesis in *Arabidopsis* Trichome Cells

Based on the findings that the composition of trichome surface wax differs from that on pavement cells, it must be assumed that both cell types have autonomous wax biosynthesis machinery, and that at least some of the genes/enzymes involved differ between them. Most investigations into wax biosynthesis so far used whole tissues, including all types of epidermal cells, and thus mixtures were strongly dominated by pavement cells rather than trichomes. Therefore, findings from these whole-tissue experiments can be taken as proxies for wax biosynthesis in pavement cells, but not necessarily for trichomes. Some recent reports now add new information on wax metabolism in trichomes, revealing both commonalities and differences between both cell types. 

Firstly, promoter activity studies using GUS staining or GFP fluorescence identified cell-type-specific expression patterns of wax biosynthesis genes. The *Arabidopsis* mid-chain alkane hydroxylase enzyme MAH1, responsible for formation of the secondary alcohol and ketone products of the alkane-forming pathway, is expressed in stem pavement cells, but not in stem trichomes or guard cells [29]. Conversely, the Arabidopsis fatty acyl-CoA reductase CER4 synthesizing wax primary *n*-alcohols is expressed preferentially in leaf trichomes rather than pavement cells [30]. GUS analyses showed also time-dependent changes in expression levels of several wax biosynthesis genes. For instance, the elongase-associated protein CER2 was found expressed in trichomes and guard cells of developing leaves, but not in mature leaves [42]. Similarly, the alkane-forming decarbonylase CER1 showed strong expression in trichomes of young leaves, and its expression level decreased with leaf maturation [43]. 

Secondly, analyses of transcriptome datasets from *Arabidopsis* leaf trichome and pavement cells confirmed that both cell types have autonomous wax biosynthetic machinery. On the one hand, several wax biosynthetic genes are expressed equally in pavement cells and trichomes, including some encoding ketoacyl-CoA synthetase (KCS) components of the fatty acid elongase (FAE) complexes (KCS3, KCS4, KCS6/CER6, KCS9, KCS11-14, KCS19, and KCS20), other FAE enzymes such as the ketoacyl-CoA reductase (KCR1), the β-hydroxyacyl-CoA dehydratase (HCD/PAS2) and the enoyl-CoA reductase (ECR/CER10), as well as proteins associated with the FAE (CER2 and CER2-LIKE2), and head-group-modifying enzymes, such as the alkane-forming reductase (CER3) and decarbonylase (CER1), and the alcohol-forming reductase (FAR3/CER4) [6,38]. Together, these genes are known to encode a full complement of wax biosynthetic enzymes, and both pavement and trichome cells, thus, likely harbor the entire machinery required to form major wax constituents, such as aldehydes, alkanes, and primary *n*-alcohols. 

On the other hand, further homologs of the genes listed above were found expressed differentially between pavement cells and trichomes. Particularly, *KCS2/DAISY* and *KCS16* were expressed, albeit weakly, only in developing trichomes, while *KCS1*, *KCS5/CER60*, *KCS8*, and *KCS10* had especially high expression signals in developing trichomes. Similarly, homologs of other enzymes associated with the FAE (*KCR2* and *CER2-like1/CER26*) and with head group modification (*CER1-like1*) were highly expressed only in developing trichomes. Finally, the sole homologs of genes encoding two other head group modification enzymes, the mid-chain hydroxylase (*MAH1*) and the wax ester synthase (*WSD1*), were also expressed preferentially in trichomes.

The KCS enzymes are known to confer chain length specificity to the FAE and, thus, to dictate overall chain length profiles of wax mixtures. Therefore, the finding that several *Arabidopsis KCS* genes are expressed preferentially in trichome cells was noteworthy, since *Arabidopsis* trichomes had also been reported to contain relatively high amounts of especially long-chain wax constituents (C_35_ and C_37_). Previously, nothing was known about the enzymatic machinery involved in elongating fatty acyl precursors beyond C_34_, and several of the KCS homologs in *Arabidopsis* had not been characterized. From these candidates, KCS16 was recently selected based on its trichome-specific expression. Detailed biochemical and molecular genetic investigations revealed that *ksc16* loss-of-function mutants were depleted of C_35+_ products in trichome and pavement cell waxes, whereas expression of KCS16 in yeast and ectopic overexpression in *Arabidopsis* resulted in accumulation of C_36_ and C_38_ fatty acids [7]. Together, these findings showed that KCS16 is the sole enzyme catalyzing the elongation of C_34_ to C_38_ acyl-CoAs in *Arabidopsis* leaf trichomes and that it is, thus, crucial for the trichome-specific formation of especially long-chain wax compounds. Overall, the characterization of KCS16 illustrates how the cell-type-specific composition of trichome wax results from differential expression of a dedicated enzyme which is homologous to ubiquitous wax biosynthesis enzymes, but has a distinct product profile. 

It is worth noting that the expression patterns of certain wax biosynthesis genes did not match wax compositional differences between trichomes and pavement cells. For instance, microarray and GUS analyses showed that the *CER4* gene, encoding the fatty acyl-CoA reductase responsible for the formation of wax primary *n*-alcohols, was expressed mostly in leaf trichomes rather than pavement cells [6,30]. However, diverse chemical analyses (of *Arabidopsis* trichomes and intact leaves, including ontogenetic time course experiments, see above) unambiguously showed that primary *n*-alcohols are present in cuticular waxes of both cell types. This seeming contradiction might be explained on the one hand by relatively high detection limits of the involved microarray experiments, and by highly-sensitive GUS staining of trichomes on the other. Even a low level of expression in pavement cells over relatively long spans of pavement development might result in sufficient enzyme activity to account for the alcohol products found on the pavement cells. Indeed, non-negligible *CER4* expression in *Arabidopsis* leaves was reported from qRT-PCR experiments [2], thus, highlighting the need to test expression data beyond microarray analyses.

In summary, all the recent studies involving transcriptome and promoter analyses confirmed that trichomes have a complete set of wax biosynthesis genes enabling autonomous wax biosynthesis. However, beyond the ubiquitous complement of wax biosynthesis genes shared between all epidermis cells, trichomes may contain (at least some) unique enzymes extending their wax metabolic pathways. Not only the expression of the genes encoding these trichome-specific enzymes differs between trichomes and pavement cells, but also the expression of other, ubiquitous wax biosynthesis enzymes. 

## 4. Extra-Long Compounds in the Wax Mixtures of Diverse Plant Species 

All the evidence discussed so far shows that *Arabidopsis thaliana* trichomes have distinct wax composition from neighboring pavement cells, due to also distinct biosynthetic mechanisms. To further understand the evolution and possible eco-physiological functions of trichome waxes, it would be interesting to know whether such cell-type-specific wax compositions and biosynthesis also occur in other species. However, only little is known about the composition of cuticular waxes covering trichomes of other plant species. 

There is scattered, indirect evidence that cuticular waxes covering trichomes of species other than Arabidopsis differ from those on respective pavement cells. For example, the leaves of *Puccinellia tenuiflora* and *Oryza sativa* are known to have epicuticular wax crystals on their pavement cell surfaces, but not on adjacent trichomes [44], indicating wax compositional differences between both epidermis cell types analogous to *Arabidopsis*. Many studies addressed the total chemical compositions of glandular trichomes, but did not investigate the trichome waxes specifically [45,46,47]. In one exceptional investigation, the trichomes isolated from peach fruit were analyzed and found to have cuticular wax consisting mainly of alkanes (92%), with chain lengths ranging from C_22_ to C_34_ [48]. In contrast, wax on accompanying pavement cells comprised several compound classes, with only 72% alkanes, and chain length profiles (C_23_–C_29_) lacking the extremely long homologs. The differences between trichome and pavement wax compositions on peach fruit, thus, strongly resemble those reported for *Arabidopsis* leaf trichomes. 

Unfortunately, the trichome waxes of no other species have been investigated to date, and it remains unclear whether cell-type-specific wax compositions similar to those of *Arabidopsis* and peach exist elsewhere. Further studies into the wax compositions of both non-glandular and glandular trichomes of diverse taxa would be of great interest to better understand the specific function of cuticles lining these special epidermis cells.

Conversely, wax compounds with extra-long hydrocarbon chains similar to those on *Arabidopsis* trichomes had previously been described in the bulk wax mixtures (extracted from whole organs without discriminating between epidermal cell types) of diverse other plant species (Table 2). It is interesting to now compare the occurrence of such C_35+_ aliphatics in diverse taxa growing in various habitats, as a backdrop for future investigations into their formation and function, possibly also in the context of trichome-specific accumulation. 

Extra-long hydrocarbon chains were typically encountered in relatively small amounts accompanying much larger quantities of the C_26_–C_34_ ubiquitous compounds in bell-shaped homolog distributions. Since trace amounts of C_35+_ wax compounds were detected in fairly diverse analyses, similar, small quantities of them may be surmised in other species as well, but some wax analyses may have failed to detect longer homologs due to instrument settings with limited sensitivity. The occurrence of very low amounts of compounds at the high end of the homolog distribution suggested that they are formed merely as by-products of the normal wax biosynthesis machinery rather than through dedicated processes specific to their chain lengths. Characterization of enzymes involved in wax precursor elongation in respective species may reveal whether, in these cases, single FAEs indeed form both the ubiquitous chain lengths and the C_35+_ homologs [2,6,23,39,49,50,51,52,53,54,55,56,57,58,59,60,61,62,63,64,65,66,67,68,69,70,71].

However, some plant species tend to accumulate relatively high amounts of extra-long hydrocarbon chains, raising the question whether in these cases C_35+_ products are made by dedicated elongase complexes. Among these species, two main distinct chain length distribution patterns can be observed: (1) In some species, the overall homolog profiles peaked at relatively long chains, such as C_33_ or even C_35_ and, thus, the further accumulation of substantial quantities of C_35+_ wax compounds fell within bell-shaped distributions that are as narrow as those of other species but, overall, shifted towards longer chain lengths. Such distributions were observed, for example, in the wax mixtures of *Austrocedrus chilensis* [58], *Pleioblastus chino* [60], *Paspalum notatum* [61], or *Cryptomerica japonica* [67]. (2) In other species, substantial amounts of C_35+_ compounds occurred within chain length profiles peaking at C_29_, and thus revolving around rather normal chain lengths but with a characteristically broad spread. Such distributions were observed in the wax mixtures of *Phalaris aquatica* [60], *Opuntia dillenii* [66], or *Bambusa dendrocalamus* [64]. Both these chain length profile types involving the accumulation of relatively high C_35+_ compound amounts are of note for further studies into wax biosynthesis. It will be interesting to characterize the KCS enzymes involved, as well as other proteins associated with the FAE complexes containing them, to understand how the chain length shifts and/or the broadening of the homolog distribution are effected. Of note, for some of the species in which C_35_ and C_37_ compounds were identified, genomic sequence data are available (*Brassica* spp., *Pisum sativum*, *Triticum aestivum*, *Lupinus angustifolius*, and *Zea mays*), which will allow further investigation of biosynthesis and function of extra-long compounds beyond *Arabidopsis*.

Beyond the machineries generating especially long acyl precursors, it will be interesting to also study the chain length specificities of the enzymes that catalyze reactions by which the acyl precursors are modified into final wax products such as alkanes and alcohols. Interesting candidate species for this purpose may be selected based on prior wax composition reports, and comprehensive cuticular wax analyses including all major compound classes are required for this. However, of those studies reporting C_35+_ wax compounds, relatively few have provided complete wax profiles. Most interestingly, they suggest that extra-long wax constituents may be limited to certain compound classes, in most cases to alkanes (Table 2). The chain length range of other compound classes, such as aldehydes or primary *n*-alcohols, tended to be shorter (usually between C_24_ and C_34_). Only rarely were longer chain lengths also described for other compound classes, such as the C_29_–C_35_ secondary alcohols in *Rosa canina* leaf wax [61].

Unfortunately, many other studies focused on analyzing alkanes, without reporting chain length profiles of other wax compound classes accompanying them. It is, hence, impossible to assess whether indeed the wax biosynthesis pathways leading to wax compound classes other than alkanes may discriminate against the exceptionally long-chain intermediates. Therefore, comprehensive analyses of diverse plant species detailing the quantities of C_35+_ constituents of all wax fractions are needed in the future.

In some plant species, the C_35+_ wax alkanes occurred together with alkenes that also had exceptionally long chains. For example, *Arabidopsis* leaf wax contained not only C_23_–C_37_ alkanes, but also exceptionally long alkenes, which however were restricted to a relatively narrow range from C_33_ to C_37_ (Figure 5) [2,6]. Similar alkenes were also reported for other species, including C_23_ to C_35_ alkenes in *Hordeum vulgare* spikes [72], a broad distribution around C_29_ in *Rosa damascena* flowers [73], and chain lengths up to C_35_ or C_37_ in cucumber fruits and stems [74], barley leaves [72], tomato fruit [75], maize pollen [76], or olive oil [77]. 

Although C_35+_ wax constituents have been reported for diverse plant species, the literature on these compounds remains patchy. The C_35+_ compounds have been identified in several species of Poaceae, Cactaceae, or Cupressaceae, and of diverse other families as well (Table 2). Thus, based on the relatively few comprehensive analyses that positively identified such compounds, their distribution across diverse taxa can hardly be assessed. It appears likely that they have been over-looked in many plant species, due to difficulties with detection by GC-MS, and that they are occurring more widely than previously thought. It is interesting to note that, even within the limited number of species where they have been detected to date, many are native to subtropical climates. Clearly, this preliminary observation will have to be corroborated by wax analyses of many more plant species from diverse habitats. Such broader surveys may be used to search for correlations between the relative amounts of C_35+_ compounds and select parameters in the growth conditions of respective species, to test for possible adaptive advantages conferred by the C_35+_ wax compounds in certain climates. 

It is also interesting to note that extra-long wax compounds were mostly identified in leaf waxes so far. However, the large majority of wax studies to date focused on leaves, and the wax mixtures on other organs, thus, cannot be compared adequately. Therefore, it is not clear whether the C_35+_ compounds indeed accumulate preferentially on certain organs, and particularly on leaves. On the one hand, *Arabidopsis* may serve as a point in case, as extra-long compounds were detected in its leaf (mainly trichome) waxes but not in the wax mixtures covering most other organs. On the other hand, in other species such as *Papaver somniferum* [59], *Miscanthus sinensis* [60], *Lotus corniculatus*, and *Trifolium repens* [61], extra-long wax compounds were also identified on stem, fruit, and inflorescence surfaces (Table 2). 

Finally, the recent findings that *Arabidopsis* trichome waxes contain relatively high amounts of C_33_–C_37_ alkanes raise the question whether the C_35+_ compounds in other species may also reside mainly, if not entirely, on trichome surfaces. To answer this question, previous reports on the occurrence of extra-long wax constituents may be integrated with further studies mentioning the presence of trichomes on relevant organs of respective species. Interestingly, many of the plant species known to have C_35_ or C_37_ alkanes lack trichomes, suggesting that extra-long wax constituents may be characteristic constituents of pavement cells as well. Unfortunately, for some species with C_35+_ wax compounds, there is no information on the presence or absence of trichomes available. In the future, comprehensive micro-morphological characterizations of all plant surfaces are needed in parallel with chemical analyses of their cuticular wax mixtures. Ideally, wax mixtures on the trichomes of diverse species should be analyzed directly using the methods recently established for direct investigation of *Arabidopsis* trichome surfaces, to search for differences in wax composition between pavement and trichome cells, and to test whether C_35+_ compounds tend to accumulate in trichome waxes of diverse species. 

## 5. Possible Functions of Extra-Long-Chain Compounds in Trichome Wax

The findings that trichomes (at least in *Arabidopsis*) have distinct wax biosynthesis machineries and, therefore, compositions raise interesting questions regarding trichome surface properties and functions. However, there is currently only very little evidence to answer such questions on the possible adaptive benefits of trichome waxes in general, and of extra-long wax compounds in particular. Hence, it may at this point only be speculated how the presence of C_35+_ constituents may affect the physical structure and, therefore, the physiological properties of respective wax mixtures.

Based on the generally accepted models for the physical structures of wax mixtures, it seems very plausible that the presence of extra-long wax constituents will have significant effects on the melting behaviour and the crystallinity, two parameters defining wax properties. Firstly, the C_35+_ wax compounds have melting points higher than those of the ubiquitous, shorter homologs, and admixtures of the extra-long chains will therefore affect the melting characteristics of the wax. The melting ranges of plant wax mixtures are known to vary drastically, depending on composition, with melting start temperatures from 40 °C to 75 °C [40,78,79] and, thus, possibly in the range of ambient temperatures in certain habitats. The presence of longer homologs will increase the percentage of wax molecules remaining in the solid state at these temperatures and may, thus, serve to keep the cuticle structure intact in especially hot micro-environments.

Secondly, the presence of especially long wax compounds will affect the packing of molecules within the complex solid-state wax mixtures. However, it is currently not clear whether the accumulation of C_35+_ compounds would enhance or impede the water barrier properties of the wax mixtures. Whether such admixtures would have positive or negative effects might depend on their concentration as well as the overall chain length distribution [80]. On the one hand, small amounts of C_35+_ compounds may be expected to merely broaden the chain length distribution and lead to increased mismatches in the side-by-side packing of shorter and longer homologs within the wax structure. The resulting local disorder would effectively reduce the overall crystallinity of the wax mixture, facilitating access for water molecules and thus negatively affecting the water barrier properties of the wax mixture. On the other hand, a sufficient admixture of C_35+_ compounds could also lead to (partial) phase separation, generating domains within the wax where higher homologs are concentrated and, consequently, both the overall crystallinity and the barrier properties of the mixture may be increased. 

Finally, other properties might be also, or even more, relevant for the waxes covering trichomes. Due to their extreme architecture and position, the trichomes are exposed to stresses that are either more severe or altogether different from those of pavement cells. Of particular importance in this context, trichomes must be exposed to mechanical stress, and their surface structures must, therefore, be relatively flexible to remain functional throughout various movements and upon contact [5]. It is not clear in how far the special chemical composition of the trichome waxes found so far may affect their mechanical properties, and in how far they may be suited to withstand this particular stress. 

## 6. Conclusions

It has recently been found that the cuticular wax covering *Arabidopsis* trichomes differs from those on adjacent pavement cells, mainly by containing C_35+_ alkanes and alkenes derived from respective C_36_ and C_38_ acyl-CoA derivatives. These extra-long precursors are formed by elongation catalyzed by FAE complexes involving KCS16, a condensing enzyme preferentially expressed in trichomes. Thus, it is now established that *Arabidopsis* trichomes have distinct surface composition due to autonomous wax biosynthesis machinery involving many of the same genes as pavement cells, but also additional elements that are trichome-specific. It seems very likely that trichomes on other species as well have wax compositions and biosynthesis apparatuses distinct from the neighboring pavement cells. However, direct evidence is required for detailed comparisons between cell types and species, to assess possible commonalities and differences and, thus, to understand how certain wax compounds may contribute to special wax functions on trichome surfaces. 

It will also be interesting to investigate in how far the waxes covering trichomes and pavement cells differ from those on the third type of epidermis cells, the guard cells. While it has not been possible to analyze the wax compositions of pavement and guard cells directly so far, there is some indirect evidence that guard cells may have a distinct wax composition. For example, differences in UV-induced fluorescence were observed between guard and other epidermal cells, possibly caused by wax-bound phenolic compounds or a thicker cuticular wax layer on guard cells. Accordingly, wax removal led to decreased fluorescence intensities from guard cells of *Olea europaea*, *Vicia faba*, and *Triticum aestivum* leaves [81]. In a separate study, the *Arabidopsis HIGH CARBON DIOXIDE* (*HIC*) gene encoding a KCS was found expressed exclusively in guard cells [82], and *hic* mutants, as well as the *cer1* and *cer6* wax biosynthesis mutants had significant increases in stomatal frequencies [82]. While these findings clearly show that cuticular wax composition influences stomata development, it remains to be determined whether, conversely, guard cell waxes may also be distinct from those on pavement cells. Unfortunately, the differences in stomatal frequencies in respective mutants were not sufficient to interpret them in terms of possibly concurring differences in wax composition. Instead, other mutants with dramatic alterations in stomata density will have to be used for comparative analyses of respective wax mixtures, to enable inferences on guard cell surface composition. Several *Arabidopsis* mutants with increased stomata density were described previously, including *tmm* [83,84,85], *sdd1* [86,87], and *yda* [88,89]. Similarly, Arabidopsis lines overexpressing *EPIDERMAL PATTERNING FACTOR* (*EPF*) genes also have increased density of stomata on the abaxial side of the leaves, while *epf* mutants are lacking stomata almost completely and have an increased number of pavement cells instead [89,90]. Chemical analyses of the cuticular waxes on these *Arabidopsis* lines with vastly differing stomata numbers may well hold the answer to the question on guard cell wax autonomy and function.

## Figures and Tables

**Table 1:** Trichome, guard cell, and pavement cell surface areas and cell numbers on adaxial *Arabidopsis* leaves (adapted from [2]).

Projected Surface Area of Blade (mm^2^)	Number of			Surface Area of	
	Trichomes (Blade^−1^)	Guard Cell Pairs (Blade^−1^)	Pavement Cells (Blade^−1^)	Pavement Cells (μm^2^ cell^−1^)	Trichome Cells (μm^2^ cell^−1^)
138	72	10366	29602	4646	40000

**Table 2:** Survey of plant species reported to contain cuticular wax compounds with extra-long hydrocarbon chains (C_35+_). Chain length ranges, most abundant chain lengths (C max.), and relative abundances of C_35+_ compounds within respective compound classes (+: 0–1%; ++: 1–5%; +++: 5–10%; ++++: >10%) are shown. Comprehensiveness of respective chemical analyses is indicated by information which compound classes were included in the analyses. As additional information, organs from which extra-long compounds were extracted are given, the occurrence of trichomes on respective tissues is assessed where possible, and climatic zones are listed for each species.

Plant Species	Family	Tissue	Analyzed Compound Classes	Compound Class	C max.	Carbon Chain Length Range	Abundance of C36/C35	Abundance of C38/C37	Climatic Zone	Trichomes	Reference
*Euphorbia characias*	Euphorbiaceae	Leaves	Complete wax profile	Alkanes	31	C19–C37	+	+	Temperate	N/A	[48]
				Aldehydes	31	C24–C36	+	N/A			
*Euphorbia cyparissias*				Alkanes	31	C19–C37	++	+			
				Aldehydes	31	C24–C36	+	N/A			
*Euphorbia lathyris*				Alkanes	31		++	+			
				Aldehydes	31	C24–C36	+	N/A			
*Euphorbia niccaensis*				Alkanes	31	C19–C37	+	+			
*Euphorbia peplus*				Alkanes	31	C19–C37	+	N/A			
*Austrocedrus chilensis*	Cupressaceae	Leaves	Alkanes only	Alkanes	33	C21–C37	++++	+	Temperate to Subtropical	N/A	[57]
*Eschscholzia california*	Papaveraceae	Leaves	Complete wax profile	Alkanes	29	C21–C37	+	N/A	Temperate	N/A	[58]
*Papaver orientale*		Leaves			29	C21–C35	+	N/A			
*Papaver somniferum*		Capsules			29	C21–C37	+	N/A			
*Miscanthus sinensis*	Poaceae	Leaves	Alkanes only	Alkanes	31	C25–C35	+++	N/A	Temperate	Yes [49]	[59]
		Senescent leaves			31		+++	N/A			
		Stems			31		++++	N/A		No [49]	
		Inflorescence			31		++	N/A		N/A	
*Pleioblastus chino*		Leaves			31		++++	N/A		Yes [50]	
*Sasa nipponica*		Leaves			31		+++	N/A		N/A	
		Senescent leaves			31		+++	N/A			
*Zoysia japonica*		Leaves			31		++	N/A		N/A	
		Senescent leaves			31		++++	N/A			
*Austrodanthonia pilosa*	Poaceae	Leaf blades	Alkanes, prim. alcohols	Alkanes	31	C25–C35	++	N/A	Temperate	N/A	[60]
*Austrodanthonia racemosa*					33		+	N/A			
*Axonopus fissifolius*		Shoot			33		++++	N/A	Subtropical		
*Bothriochloa macra*		Leaves			27		++	N/A	Temperate		
*Bromus catharticus*		Shoot			29		++	N/A			
*Chloris gayana*		Leaves			31		++	N/A	Subtropical	N/A	
		Shoot			33		++++	N/A			
*Cynodon dactylon*					33		+++	N/A		Yes [51]	
*Digitaria didactyla*					33		++++	N/A		N/A	
*Elymus scaber*		Leaves			31		++	N/A	Temperate	N/A	
*Festuca arundinacea*		Shoot			31		+	N/A		N/A	
*Imperata cylindrica*					31		+++	N/A	Subtropical		
*Lotus corniculatus "Prostate"*		Shoot			29		+	N/A	Temperate	No [52]	
*Lotus pedunculatus cv. Maku*					29		+	N/A		Yes [52]	
*Microlaena stipoides*		Leaves			31		++	N/A		N/A	
*Paspalum dilatatum*		Shoot			33		++++	N/A	Subtropical		
*Paspalum notatum*					35		++++	N/A			
*Pennisetum clandestinum*					35		++++	N/A		N/A	
*Phalaris aquatica*					29		+++	N/A	Temperate	N/A	
*Setaria anceps*					27		++	N/A	Subtropical		
*Sporobolus indicus cv. Major*					33		++++	N/A		N/A	
*Themeda australis*		Leaves			31/33		+++	N/A	Temperate		
*Trifolium repens*	Fabaceae	Shoot			31		+	N/A		Yes [53]	
*Vulpia myuros*	Poaceae				31		+	N/A		N/A	
*Brassica spp.*	Brassicales	Leaves	Alkanes only	Alkanes	N/A	C17–C35	N/A	N/A	Temperate	Yes [54]	[37]
*Pisum sativum*	Fabaceae									N/A	
*Rosa canina*	Rosaceae	Leaves	Complete wax profile	Sec. alcohols	N/A	C29–C35	+	N/A	Temperate	N/A	[61]
*Wollemia nobilis*	Araucariaceae	Leaves	Alkanes only	Alkanes	N/A	C33–C35	N/A	N/A	Temperate	N/A	[62]
*Bambusa bambusa*	Poaceae	Leaves	alkanes only	Alkanes	31/33	C23–C35	+++	N/A	Tropical	N/A	[63]
*Bambusa dendrocalamopsis*					29		+	N/A			
*Bambusa dendrocalamus*					29		++				
*Alternanthera dentata*	Amaranthaceae	Leaves	Alkanes only	Alkanes	29	C22–C35	++	N/A	Subtropical	N/A	[65]
*Alternanthera versicolor*					31	C18–C35	+	N/A			
*Araucaria cunninghamii*	Araucariaceae				31	C22–C35	++	N/A			
*Bothriochloa ischaemum*	Poaceae				31	C14–C35	++	N/A		N/A	
*Caryota mitis*	Arecaceae				31	C20–C35	+	N/A		N/A	
*Cinnamomum burmannii*	Lauraceae				31	C22–C35	+	N/A			
*Codiaeum variegatum*	Euphorbiaceae				33	C22–C35	++	N/A			
*Euphorbia trigona*					33	C24–C37	++	N/A			
*Holmskioldia sanguinea*	Lamiaceae				35	C22–C37	++++	N/A			
*Hylocereus undatus*	Cactaceae				33	C18–C37	+++	N/A			
*Imperata cylindrica*	Poaceae				31	C14–C35	+++	N/A			
*Kigelia africana*	Bignoniaceae				31	C24–C35	+++	N/A			
*Opuntia dillenii*	Cactaceae				29	C23–C36	++++	N/A		N/A	
*Osmanthus fragrans*	Oleaceae				31	C24–C35	++	N/A		N/A	
*Pistia stratiotes*	Araceae				31	C24–C37	+	N/A		Yes [55]	
*Swietenia mahagoni*	Meliaceae				31	C24–C35	+	N/A		N/A	
*Zoysia japonica*	Poaceae				33	C14–C35	+++	N/A		N/A	
*Aspidosperma spp.*	Apocynaceae	Leaves	Alkanes, alkanols	Alkanes	33	C29–C35	+++	N/A	Tropical	N/A	[66]
*Cryptomeria japonica*	Cypressaceae		fatty acids		33	C33–C35	++++	N/A	Temperate		
*Juniperus osteosperma*					33	C29–C35	+++	N/A	Temperate		
*Manilkara spp.*	Sapotaceae				33	C31–C35	+++	N/A	Tropical		
*Arabidopsis thaliana*	Brassicaceae	Leaves ^1^	Complete wax profile	Alkanes	29	C25–C35	+	N/A	Temperate	Yes	[21]
*Miscanthus sinensis*	Poaceae	Leaves	Alkanes, fatty acids	Alkanes	31	C25–C37	+	+	Temperate	Yes [52]	[67]
*Lupinus angustifolius*	Fabaceae	Leaves	Complete wax profile	Alkanes	N/A	C23–C37	N/A	N/A	Temperate	N/A	[68]
*Triticum aestivum*	Poaceae				31					N/A	
*Olea europaea*	Oleaceae	Leaves ^1^	Alkanes only	Alkanes	29	C27–C35	+	N/A	Mediterranian	N/A	[69]
		Olive oil			25	C21–C35	+	N/A		N/A	
*Arabidopsis thaliana*	Brassicaceae	Young/mature	Complete wax profile	Alkanes	31	C27–C37	+	+	Temperate	N/A	[2,7]
		leaves		Alkenes	35	C35–C37	+	+			
*Arabidopsis thaliana*	Brassicaceae	Leaves ^1^	Complete wax profile	Alkanes	31	C27–C37	+	+	Temperate	N/A	[6]
				Alkenes	35	C35–C37	+	+			
		Leaf trichomes ^1^		Alkanes	31/33	C27–C37	+	+			
				Alkenes	35	C33–C37	+	+			
*Ludwigia octovalvis*	Onagraceae	Young leaves	Alkanes, fatty acids	Alkanes	23	C15–C35	+	N/A	Tropical	Yes [56]	[70]
		Mature leaves			23		+	N/A			

N/A: no information available; ^1^ other organs were included in the analyses, but were found to lack C_35+_ compounds.
